# Microbiota-Mediated Immune Regulation in Atherosclerosis

**DOI:** 10.3390/molecules26010179

**Published:** 2021-01-01

**Authors:** Sahar Eshghjoo, Arul Jayaraman, Yuxiang Sun, Robert C. Alaniz

**Affiliations:** 1 Department of Microbial Pathogenesis and Immunology, College of Medicine, Texas A&M University Health Science Center, Bryan, TX 77807, USA; eshghjoo@tamu.edu; 2 Artie McFerrin Department of Chemical Engineering, Texas A&M University, College Station, TX 77840, USA; arulj@mail.che.tamu.edu; 3 Department of Nutrition, Texas A&M University, College Station, TX 77843, USA

**Keywords:** microbiota, atherosclerosis, innate immunity, microbiome metabolites, macrophage

## Abstract

There is a high level of interest in identifying metabolites of endogenously produced or dietary compounds generated by the gastrointestinal (GI) tract microbiota, and determining the functions of these metabolites in health and disease. There is a wealth of compelling evidence that the microbiota is linked with many complex chronic inflammatory diseases, including atherosclerosis. Macrophages are key target immune cells in atherosclerosis. A hallmark of atherosclerosis is the accumulation of pro-inflammatory macrophages in coronary arteries that respond to pro-atherogenic stimuli and failure of digesting lipids that contribute to foam cell formation in atherosclerotic plaques. This review illustrates the role of tryptophan-derived microbiota metabolites as an aryl hydrocarbon receptor (AhR) ligand that has immunomodulatory properties. Also, microbiota-dependent trimethylamine-*N*-oxide (TMAO) metabolite production is associated with a deleterious effect that promotes atherosclerosis, and metabolite indoxyl sulfate has been shown to exacerbate atherosclerosis. Our objective in this review is to discuss the role of microbiota-derived metabolites in atherosclerosis, specifically the consequences of microbiota-induced effects of innate immunity in response to atherogenic stimuli, and how specific beneficial/detrimental metabolites impact the development of atherosclerosis by regulating chronic endotoxemic and lipotoxic inflammation.

## 1. Introduction

Cardiovascular disease (CVD) is a complex human disease that restricts blood flow in the heart and blood vessels [1]. Atherosclerosis is a major form of CVD, symbolized by excess buildup of arterial plaque (atheroma) along the arterial wall [2]. The arterial plaque in atherosclerosis is composed of lipids (cholesterol and fatty acids), debris, fibrotic material, macrophages (Macs), dendritic cells (DCs), and some other host immune cells [3]. Within the atherosclerotic plaques, Macs polarize to a pro-inflammatory state which ingests and degrade debris and lipids, promoting the formation of foam cells in the plaques, leading to the adverse effects of restricted blood flow [4].

Gut microbiota refers to microorganisms (with gene makeup distinctive from the host) living in the GI tract, which produce various unique metabolites. Metabolites refer to small molecules that result from metabolic processes, produced endogenously by the host and by microorganisms’ processing of dietary compounds. Identification of a microbiome profile in the GI tract and the functional determination of microbiome-derived metabolites are very important for health and disease [5,6,7]. A study demonstrated that tryptophan-derived compounds are depleted in the GI tract and the circulation of germ-free mice, indicating these tryptophan metabolites are dependent on the microbiota [6]. Indole is an important beneficial metabolite produced from tryptophan; it has been shown that indole is not detected in the cecal tissue of germ-free mice [6].

**Figure 1:**
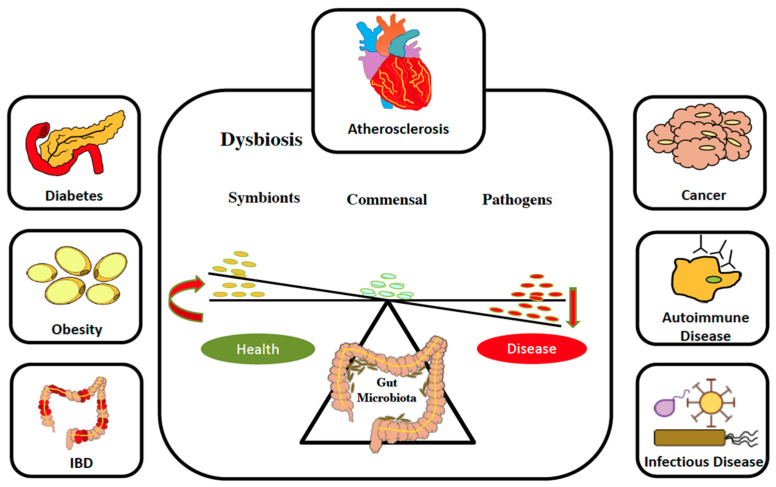
The microbiota is important for overall health. The balance between health and disease is regulated by microbiota in many ways. Microbiota in equilibrium is linked with homeostasis; when it is perturbed, it leads to dysbiosis and diseases.

There is now an increasing appreciation that the microbiota is an essential partner in overall gut homeostasis and host health. Furthermore, when the microbiota is perturbed by environmental or dietary stresses (referred to as dysbiosis), it can lead to increased inflammation and altered metabolism in the host [7] (Figure 1). There is a wealth of compelling evidence that the microbiota is linked with multiple complex diseases, including CVD [8]; however, our understanding of the mechanisms of how the microbiota affect CVD is limited. Perhaps the most specific example of the link between the microbiota and CVD is the production of trimethylamine-*N*-oxide (TMAO), the oxidized form of trimethylamine (TMA), a microbiota-dependent detrimental metabolite derived from diets rich in phosphatidylcholine, choline, and L-carnitine, associated with a significantly increased risk of atherosclerosis [9]. Although a number of microbiota-derived metabolites have been identified and studied, the full array of activities for most individual metabolites has not been completely established, further research is needed to better understand the properties and functions of the metabolites, the microbe(s) that produce them, the cellular and molecular targets, and their roles in health and disease. Thus, in this review, we discuss that the microbiota promotes atherosclerosis by the production of specific beneficial (e.g., Indole) and detrimental (e.g., Indoxyl Sulfate, TMA/TMAO) metabolites [10], and their impacts on the development of atherosclerosis in obese patients by regulating chronic endotoxemic/lipotoxic inflammation and metabolic functions.

The published data has investigated the impact of the microbiota as a rich source of potent immunomodulatory metabolites derived from tryptophan (Trp) [11]. In particular, the primary microbiota-derived Trp-metabolite indole (C8H7N), a potent endogenous ligand for the aryl hydrocarbon receptor (AhR), regulates gut inflammation, and microbiota dysbiosis [12]. We specifically discussed the inflammatory and metabolic responses of pro-atherogenic phagocytes, especially macrophages and their polarization, exposed to lipotoxic compounds and their regulation by the microbiota-derived metabolites.

## 2. Microbiota-Derived Metabolites Associated with Atherosclerosis

The microbiota is recognized for its role in the production of beneficial SCFAs and their functions in the regulation of complex chronic inflammatory diseases such as atherosclerosis. A link between the microbiota and atherosclerosis emerged from studies of TMAO [13,14,15].

A significant hallmark of atherosclerosis is the accumulation of pro-inflammatory Macs and dendritic cells (DCs) in coronary arteries that respond to pro-atherogenic stimuli, such as free fatty acids (FFAs) and oxidized LDLs (oxLDLs), and the failure to digest lipids that contribute to the formation of foam cells in atherosclerotic plaques [16,17]. Mechanisms that reduce Mac/DC inflammation, increase lipid degradation, and prevent foam cell formation would all decrease atherosclerosis progression.

There is overwhelming evidence that microbiome, microbial metabolism, and microbiota-derived/dependent nutritional metabolites contribute to the pathogenesis of atherosclerosis [18]. The mechanistic links between gut microbiota and health/disease outcome are largely undefined. Indole, TMAO, and Indoxyl sulfate are among the few best-studied microbiome-derived/dependent metabolites that have been reported to have roles in the regulation of atherosclerosis [19].

### 2.1. TMAO (Trimethylamine-N-Oxide)

TMAO is a microbial dependent metabolite. It is a byproduct of microbial metabolism of L-carnitine and choline in the gut after ingestion of eggs, meat, or fish, and TMAO is directly correlated with atherosclerosis [20]. After metabolizing carnitine and choline to TMA, through the bacteria, TMA is absorbed from the gut and transferred into the circulation [21]. Then, via an enzyme named Flavin monooxygenase, TMA is oxidized into TMAO in the liver. Other than atherosclerosis, plasma levels of TMAO are increased in patients with chronic kidney diseases (CKD) and diabetes as well [22]. The reason for the TMAO increase in these diseases is still unknown. Some studies suggest that reduced clearance of TMAO by kidneys in CKD or increased TMAO metabolism levels by bacteria in the gut are among the possible reasons [23]. Other data suggest that dysbiosis is directly linked to increased serum levels of TMAO. In the CKD patients, the relative abundance of dominant bacteria changes, and the expression of the enzyme that leads to TMAO production increases [24].

**Figure 2:**
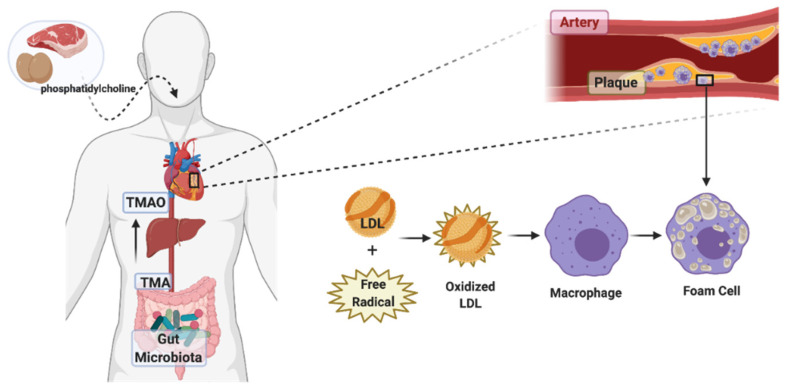
Role of trimethylamine N-oxide in atherosclerosis. Schematic pathway of phosphatidylcholine transformation to TMA and TMAO (trimethylamine N-oxide) via the gut microbiota. Dietary intake of foods like red meat and egg can alter the composition of gut microbiota. It can result in increased TMA production levels, subsequently leading to increased TMAO synthesis in the liver, eventually leading to elevated levels of oxidized LDLs and increased plaque formation. Accumulated foam cells in the plaques are lipid-laden Macs that have ingested modified lipoproteins, having a foamy appearance. In atherosclerosis, inflammatory Macs are converted into foam cells.

Rodent studies also show that TMAO, as a risk factor, enhances atherosclerosis prevalence through increasing the expression of scavenger receptors and reducing cholesterol efflux in Macs, and consequently increases levels of foam cell formation of the aforementioned Macs [25]. This proatherogenic metabolite has a positive correlation with lesion formation and development, as well as the size of the atherosclerotic plaque in arteries [26]. Atherosclerosis starts with the accumulation of foam cells in the arteries. Monocytes in the circulation penetrate the arteries where there are lesions and transform into Macs; by phagocyting modified cholesterol and lipoproteins, these Macs transform into foam cells (Figure 2). Accumulation of foam cells under the artery endothelial cells then form plaques, and the rupture of the plaque can block arteries and cause a stroke [27].

Macs regulate lipoprotein metabolism and are the key cells involved in atherosclerosis because they are the origin of the foam cells. The migration of Macs to the plaque areas and the mechanisms of foam cell transformation have very important implications. There is some evidence suggesting that TMAO is involved [28]. When the LDLs or low-density lipoproteins are oxidized by the free radicals in the arterial walls, the Macs are triggered to phagocytose these modified lipoproteins by increasing the expression of scavenger receptors such as CD36 and SRA-1 that have high sensitivity to modified lipoproteins [29]. TMAO upregulates these scavenger receptors’ expression and induces the uptake of these modified LDLs by Macs [30]. TMAO increases Macs’ migration and promotes the expression of inflammatory cytokines such as IL-6 and TNF [31].

### 2.2. Indoxyl Sulfate (I3S)

**Figure 3:**
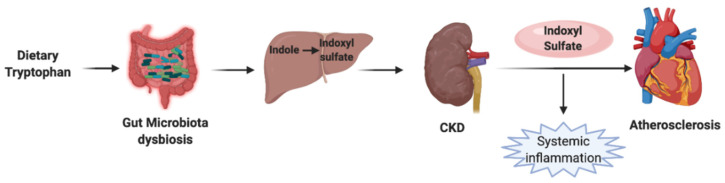
Effect of Indoxyl sulfate in atherosclerosis. Schematic presentation of the Indoxyl sulfate pathway is linked to atherosclerosis. Tryptophan is metabolized by the gut microbiota into indole, and indole is absorbed into the circulation. In the liver, indole is metabolized to indoxyl sulfate. In chronic kidney disease and dysbiosis conditions, kidneys are incapable of clearing indoxyl sulfate. This results in the accumulation of indoxyl sulfate. Systemic inflammation caused by indoxyl sulfate can cause coronary calcification and chronic cardiovascular abnormalities, eventually leading to atherosclerosis.

Indoxyl sulfate, aka, 3-indoxyl sulfate or 3-indoxylsulfuric acid (I3S), is a bacterial metabolic byproduct of dietary nutrients. I3S plays a role as a uremic toxin and a cardiotoxin [32]. A microbial enzyme, named tryptophanase, catabolizes the tryptophan to indole. Then the indole gets absorbed and converted into indoxyl sulfate in the liver. Dysbiosis and epigenetic alterations of the gut microbiota alter the amino acid metabolism and increase the levels of I3S in the serum (Figure 3) [33]. Also, the amount of tryptophanase expression in the microbiota increases to facilitate Indoxyl sulfate production in CKD and CVD patients [34], and the prevalence of atherosclerosis is higher in CKD patients [35]. Based on the epidemiology studies, CKD patients have a high risk for atherosclerosis beyond traditional risk factors, indicating the possible role of microbiota in the pathogenesis of the diseases, such as CKD and CVD.

Plasma concentration of I3S increases in atherosclerosis and CKD patients [36]. I3S has a high affinity to proteins, and therefore it cannot be removed by kidneys easily [37]. In vitro studies show that indoxyl sulfate can enhance leukocyte activation and increase their adhesion to endothelial cells and eventually cause elevated levels of oxidative stress and inflammation [38]. Furthermore, it is hypothesized that indoxyl sulfate reduces Macs’ cholesterol efflux and induces foam cell formation [39]. I3S is also related to glucose intolerance by reducing GLUT-1 expression and the hepatic LXR signaling pathway [40]. Thus, increased levels of inflammatory Macs may reflect the severity of atherosclerosis. 

Proteomics studies indicate activation of some pathways via I3S in Macs, such as the ubiquitin-proteasome pathway and Notch signaling [41]. Some studies show that membrane transport proteins such as OATP2B1 regulates the uptake of I3S in Macs [42]. I3S is an agonist for AhR, and it can increase cell proliferation of vascular smooth muscle through AhR and activation of NF-κB signaling [43]. Furthermore, I3S is known to increase ROS production [44].

### 2.3. Indole

**Figure 4:**
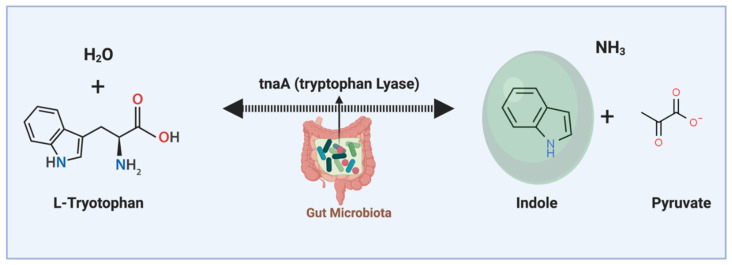
Indole is a gut microbiota-derived metabolite. Indole is produced from tryptophan amino acid through the action of the tryptophan lyase enzyme. Indole is an aromatic heterocyclic organic molecule and has a bicyclic structure. Indole has anti-inflammatory regulatory effects on intestinal epithelial cells and immune cells in the gut and distal organs (through circulation) in the body.

Indole is a gut microbiota-derived tryptophan catabolite. It is produced during the tryptophan metabolic process by the tryptophan lyase (tnaA) enzyme (Figure 4) [45]. Indole is an agonist for the aryl hydrocarbon receptor (AhR). There is a high concentration of indole in the GI tract, and it can enter the blood circulation [46]. Indole is detectable in human and mouse luminal contents at 0.1 to 4 mM concentrations and around 0.1 to 10 µM in the circulation [47]. Indole is a very small molecule, and it is easily diffusible and can directly interact with immune cells [48].

It has been reported that indole has anti-inflammatory effects [49]. Studies show that plasma levels of indole and indole derivatives are negatively correlated to advanced atherosclerosis [50]. Indole is produced by various gram-negative and gram-positive bacteria species, such as *E. coli*, *Bacteroides*, and *Clostridium* [51]. In in vitro studies have indicated that other than immunomodulatory effects, indole promotes health outcomes in intestinal epithelial cells in humans and rodents by preventing colitis induced by dextran sulfate sodium (DSS) and strengthening the epithelial barrier [52]. A study suggested the important positive role of indole supplementation to facilitate anti-inflammatory drugs [53].

Gut bacteria genomic information of the human microbiome suggests that tryptophan metabolites are the most important bioactive microbiota metabolites [54]. Studies demonstrate that in atherosclerotic patients, microbiome tryptophan synthesis takes place; consequently, plasma levels of tryptophan metabolites are reduced [55]. There are some tryptophan derivatives, such as indoxyl sulfate or indole acetate, where increased levels of these metabolites have been reported to have a direct correlation with CVD and other diseases [56]. This suggests the importance and sensitivity of the equilibrium of microbial tryptophan metabolites in the host’s gut and overall health. Molecular targets of tryptophan derivatives are still unknown, but some indoles have been reported to have modulatory effects through the AhR [57]. A study proposes the development of antibiotics to control the metabolic changes in atherosclerosis. Data indicated that diminished microbiota metabolism by antibiotics reduced tryptophan metabolism and exacerbated atherosclerosis [55]. Studying the crosstalk between metabolic changes, immune cells, and molecular targets is currently a very important topic in atherosclerosis research.

## 3. Aryl Hydrocarbon Receptor (AhR)

AhR is a ligand-activated transcription factor. AhR is activated by various endogenous and exogenous polycyclic aromatic hydrocarbon ligands [58]. AhR was first identified as a receptor for industrial toxin, n-dioxin [59]. Now, AhR is recognized as a significant mediator of immune cell activity, particularly in the GI tract [60].

AhR is involved in different cellular activities such as cell differentiation/proliferation, cytokine production, and responses to environmental toxins [61]. AhR has been suggested to have roles in the gut and immune system, such as the regulation of the intraepithelial lymphocytes (IELs) and innate lymphoid cells (ILC) in the gut [62].

AhR has been reported to regulate the induction of T-reg and Th17 [63]. The crosstalk between intestinal microbiota and immunity, and the role of AhR in antigen-presenting cells (APCs) have not been well-characterized. AhR can detect environmental signals such as dietary ligands [64]; it is also present in immune cells [64,65]. AhR is considered a candidate pattern recognition receptor sensor for immune responses driven by nutritional and microbial gut metabolites [65].

AhR exists in the cytosol, binds to the ligand, and then translocates to the nucleus via a nuclear translocator to act as a transcription factor [58,66]. In a study detecting a panel for AhR ligands in BMDCs, some ligands like I3C (indole 3-carbinol) and FICZ (6 formyl indolo carbazole) have been among ligands that induce pro-inflammatory effects in lipopolysaccharides (LPS) induced APCs, which is in contrast with the studies that suggest AhR activation has anti-inflammatory immunomodulatory effects [67]. For example, 4-n-nonylphenol, an agonist for AhR, can induce T-regs [68]. The published data suggest that AhR signaling in monocyte-derived-Macs has an important impact on the function of Macs [69]. A detailed investigation of ligands and their functional dependency on AhR is necessary to unravel the role of AhR in regulating Macs, and how it interacts with endogenous intestinal ligands.

Studies highlight that the expression of AhR is related to atherosclerosis [70], but the effects of AhR activation is dependent on the agonists, species, and cell type. For example, in ApoE-KO mice, increased AhR is linked to increased symptoms of atherosclerosis, but on the other hand, activation of AhR through indoles has modulatory effects on the reduction of CVD [70,71]. Some studies highlight the beneficial role of specific indoles in the prevention of atherosclerosis [72]. Collectively, data regarding AhR in atherosclerosis show that AhR activation has both beneficial and adverse effects depending on the different circumstances. Since the role of AhR is different in species, data from rodent studies may not be an accurate indication of its function in humans.

## 4. Metabolic Impairment, Inflammation, and Endotoxemia in CVD

Bacterial endotoxins LPS, aka lipoglycans, are macromolecules composed of an O-antigen containing polysaccharide and a lipid, joined by a covalent bond. The outer cell membrane of the gram-negative bacteria has conserved components of LPS [73]. LPS can lead to innate immunity activation and the onset of inflammatory reactions [74]. Usually, plasma endotoxin levels in patients with sepsis are at ~300 pg/mL; high levels of LPS can cause sepsis both in humans and rodents [74,75,76]. “Metabolic endotoxemia” happens when there is a low but constant level of LPS in blood circulation which can cause innate immune responses and consistent inflammation in the circulation without signs of significant infection [76].

Other than sepsis, there are other diseases that are caused by metabolic and inflammatory dysregulations, e.g., insulin resistance (IR), type 2 diabetes, and atherosclerosis [77]. Many factors in obesity (such as high levels of cholesterol, saturated fatty acids, endotoxins, etc.) can raise the levels of inflammation, reverse cholesterol transport (RCT), and reduce insulin signaling [78]. Macs are the most important cells involved in the process of upregulation and downregulation of metabolic-inflammatory responses. Macs use the induction of metabolic pathways in response to an overabundance of lipids from adipocytes, e.g., saturated fatty acids (particularly palmitate) and circulating modified lipids [79]. AMPK (AMP-activated protein kinase) and PPARs (peroxisome proliferator-activated receptors) are important regulators of metabolic and inflammatory mechanisms through modulating cellular homeostasis (80). Palmitate, a known saturated fatty acid, causes metabolic inflammation through NLRP3 or (NOD)-like receptor protein 3 inflammasome and inhibits AMPK activation [79,80]. In contrast, unsaturated fatty acids normally do not cause metabolic inflammation [81].

Studies show that high levels of LPS in the serum directly affect the onset or exacerbation of CVD, especially atherosclerosis [82]. Experiments in rodents have indicated that endotoxins accelerate the incidence and progression of atherosclerosis [83]. The LPS receptor is toll-like receptor-4 (TLR-4). Mice that are deficient for TLR-4 are more resistant to atherosclerosis development or progression [84]. It is hypothesized that when there is endotoxin alone without infection, LPS from intestinal gram-negative bacteria or nutrients can translocate from the intestinal lumen to the circulation [85]. Furthermore, increased endotoxemia levels have shown a direct correlation with increased levels of dietary fat uptake in rodents and humans [77].

Permeability of the intestinal lumen plays an important role in the severity of endotoxemia because of the high density of endotoxins in the intestine compared to the plasma [86]. Patients with CVD have increased plasma endotoxins, indicating pathological signs of luminal permeability [82]. Microbiota and the metabolites produced by the gut microbiome play an important role in the permeability of the intestinal lumen [87].

## 5. Conclusions

Since microbiota-derived or dependent metabolites have substantial beneficial or detrimental effects in gut and distal organs, cell-specific investigations are highly valuable. In terms of atherosclerosis, metabolites in macrophages have an important role in preventing foam cell formation. Other than tryptophan metabolites, different microbiota-dependent metabolites such as TMAO metabolism have been reported to have a role in atherosclerosis progression. Overall, this review underscores the importance of microbiota, their dependent metabolites, the effect of metabolites on innate immunity, and the development of atherosclerosis.

Multi-dimensional investigations, both targeted and unbiased approaches, are warranted to further elucidate the mechanistic pathways mediating the effects of microbiome-derived metabolites and to fully unravel the impact of microbiota on pathogenesis and prognosis of atherosclerosis. Both the direct effects of metabolites in the heart as well as the indirect effects via the gut-brain axis and endocrine system should be considered. Modern nanomedical techniques may be proven to be helpful in advancing the microbiome as a feasible therapeutic strategy. In addition, since age, gender, and ethnic background are known to affect disease development, future research investigations and clinical trials of metabolites should be designed to assess the influences of these factors as well.

Although studies have shown that tryptophan metabolism has an essential role in regulating atherosclerosis, the mechanistic role of the indoles through gut microbiota has not been fully unraveled. Future works need to elucidate the mechanisms of actions by which microbiota metabolites induce inflammatory or anti-inflammatory, pro-foam cell or anti-foam cell formation, and pro-autophagic or anti-autophagic functions. Additional perspectives regarding novel endogenous therapeutics that regulate Mac’s metabolism will be extremely helpful in atherosclerosis prevention and treatment.
